# 

**Published:** 1914-07

**Authors:** 


### CONTENTS.

ORIGINAL COMMUNICATIONS—

The Treatment of Nervous Diseases Following Syphilis. By Lewis M. Gaines, M. D., Atlanta, Ga., and Allen M. Bunce, M. D., Atlanta, Ga. .. 145

Pyorrhea and Its Constitutional Effects. By William Crenshaw, D. D. S., Atlanta, Ga. ...... 157

EDITORIAL .................................. 164

SELECTIONS AND ABSTRACTS ............... 166

BOOK REVIEWS ................................

